# Formulation and Evaluation of Apigenin-Loaded Hybrid Nanoparticles

**DOI:** 10.3390/pharmaceutics14040783

**Published:** 2022-04-03

**Authors:** Imran Kazmi, Fahad A. Al-Abbasi, Syed Sarim Imam, Muhammad Afzal, Muhammad Shahid Nadeem, Hisham N. Altayb, Sultan Alshehri

**Affiliations:** 1 Department of Biochemistry, Faculty of Science, King Abdulaziz University, Jeddah 21589, Saudi Arabia; fabbasi@kau.edu.sa (F.A.A.-A.); mhalim@kau.edu.sa (M.S.N.); hdemmahom@kau.edu.sa (H.N.A.); 2 Department of Pharmaceutics, College of Pharmacy, King Saud University, Riyadh 11451, Saudi Arabia; salshehri1@ksu.edu.sa; 3 Department of Pharmacology, College of Pharmacy, Jouf University, Sakakah 72341, Saudi Arabia; afzalgufran@ju.edu.sa

**Keywords:** Apigenin, breast cancer, optimization, cytotoxicity study, antioxidant activity

## Abstract

Apigenin (AGN) is a potent phytochemical with strong antioxidant and anticancer potential. But its therapeutic efficacy is limited due to its high lipophilic characteristics. Therefore, the present investigation aimed to develop AGN-loaded polymer-lipid hybrid nanoparticles (AGN-PLHNPs). Herein, we successfully developed AGN-PLHNPs and optimized them by a 33-Box-Behnken de-sign. The poly (lactic-co-glycolic acid) (PLGA; coded as F1), phospholipon 90 G (PL-90G; coded as F2), and poloxamer 188 (P-188; coded as F3) were considered as the independent factors while particle size (PS; coded as R1), entrapment efficiency (%EE; R2), and cumulative drug release (%CDR; R3) were selected as dependent responses. The average PS, %EE, and %CDR of the AGN-PLHNPs were observed in the range of 101.93 nm to 175.26 nm, 58.35% to 81.14%, and 71.21% to 93.31%, respectively. The optimized AGN-PLHNPs revealed better homogeneity (poly-dispersity index < 0.2) and colloidal stability with high zeta potential (>25 mV). It also exhibited fast release in the initial 4 h after that sustained release up to 48 h of study. Moreover, the results of both DPPH as well as ABTS assays revealed significant improvement in the antioxidant activity. Furthermore, the optimized AGN-PLHNPs exhibited enhanced cytotoxicity efficacy against MCF-7 as well as MDA-MB-231 breast cancer cell lines.

## 1. Introduction

Cancer is one of the deadliest malignant diseases that show uncontrolled and ab-normal cell division and can invade other tissues/organs. The uncontrolled growth of cancerous cells destroys the adjacent normal healthy cells/tissues and when it becomes a mass then it is called a tumor [1]. Among a variety of solid tumors, breast cancer (BC) is the most common cancer among women and one of the leading reasons for cancer-related deaths among women [2]. Globally, more than 1 million women are diagnosed with BC every year and it accounts for more than 1.6% of cancer-related mortality [3]. In the present era, chemotherapy, surgery, and radiotherapy are the main treatment options for BC. However, chemotherapy is still not completely effective in the management of advanced-stage BC. Most chemotherapeutic drugs are characterized by high lipophilicity and low water solubility and show low bioavailability thereby low therapeutic efficacy. Moreover, chemotherapeutic drugs are unable to differentiate between the cancerous and normal healthy cells and also kill normal cells during chemotherapy. Furthermore, most of the chemotherapeutic drugs represent serious adverse effects due to high doses. Therefore, site-specific chemotherapeutic drug delivery to the solid tumor can be a better approach. Drug delivery through a novel delivery carrier can overcome these challenges. Nanoparticle-based drug delivery significantly reduces the dose of chemotherapeutic drugs, decreases the dose-related toxicity, and shows better therapeutic efficacy [4,5,6]. 

Apigenin (AGN) is a natural flavonoid present in various vegetables and fruits as well as in some medicinal plants [7]. Various studies revealed that the AGN represents a variety of therapeutic potential like anticancer, anti-inflammatory, antioxidant, antidiabetic, and many more [8,9]. AGN is highly lipophilic in nature and showed very limited aqueous solubility that resulting poor dissolution that limits its clinical application [10]. 

Polymer-lipid hybrid nanoparticles (PLHNPs) are considered as the latest generation colloidal nanocarrier consisting of polymeric core-shell enveloped by lipid layer prepared from a hybrid mixture of polymer and lipid [11]. The hybrid system provides better physicochemical characteristics and can encapsulate lipophilic as well as hydrophilic drugs in their unique hybrid matrix [12]. PLHNPs combined the advantages of both lipid as well as polymeric nanocarriers and provides small particles size, better homogeneity and colloidal stability, tuneable drug release, high loading capacity, and site-specific targeting [13]. From the last decades, PLHNPs are extensively investigated for anticancer drug delivery to treat various solid tumors. Their hybrid matrix helps in the release of encapsulated drugs to the tumor site in a controlled manner to achieve significant tumor inhibition. In addition, the small size and stability in the biological system also improve the overall therapeutic efficacy of encapsulated drugs [14,15,16].

In this investigation, AGN-encapsulated PLHNPs were developed and optimized by the three-factors, three-level Box–Behnken design (3^3^-BBD) and evaluated for various physicochemical characteristics. The optimized AGN-PLHNPs were evaluated to determine their colloidal stability and dissolution profiles. The optimized AGN-PLHNPs were evaluated for antioxidant and cytotoxicity activity by MTT assay on MCF-7 and MDA-MB-231 cells.

## 2. Materials and Methods

### 2.1. Materials

Apigenin (AGN) and a dialysis tube (MWCO: 10 and 12 kDa) were procured from Sigma-Aldrich, St. Louis, MO, USA. Poly(D, L-lactide-co-glycolide) (PLGA 50:50; polymer) was duly provided as a gift sample by Evonik India Pvt Ltd., Mumbai, India. Phospholipon 90G (Lipid; PL-90G) was duly gifted by Lipoid GmbH, Ludwigshafen, Germany. Polox-amer-188 (P-188; Surfactant) was kindly gifted by BASF, Mumbai, Maharashtra, India. MDA-MB-231 and MCF-7 cancer cells were procured from National Centre for Cell Science (NCCS), Pune, Maharashtra, India. All other chemicals with high purity were purchased from Merck, Mumbai, India).

### 2.2. Box–Behnken Statistical Design

In the successful development of PLHNPs, different factors such as the concentration of polymer, lipid and surfactant play important roles that affect particle size (PS), entrapment efficiency (%EE), and cumulative drug release (%CDR). Therefore, in present investigation, the concentration of polymer, lipid, and surfactant was optimized by 33-BBD by using Design-Expert^®^ software (Design-Expert, V-11.1.0.1, State-Ease Inc., Minneapolis, MN, USA) for the successful development of PLHNPs [17]. For the optimization of AGN-PLHNPs by 3^3^-BBD, the concentrations of PLGA (coded F_1_; 7–11 mg/mL), PL-90G (coded as F_2_; 5–9 mg/mL), and P-188 (coded as F_3_; 0.75–1.25%) were selected as independent factors at 3 different levels, high (coded as “+1”) medium (coded as “0”), and low (coded as “−1”), respectively, as shown in Table 1. On the other hand, the particle size (PS; coded as R_1_), entrapment efficiency (%EE; coded as R_2_), and cumulative drug release (%CDR; coded as R_3_) of the AGN-PLHNPs were considered to be the responses.

After following the 3^3^-BBD, 15 AGN-PLHNP formulation compositions with 3 repetitions were obtained. After this, all 15 AGN-PLHNPs were formulated according to the composition and the actual values were included in the design, as shown in Table 2. After fitting the data of all the formulations, different statistical models such as linear, 2-F1, and quadratic ones were analyzed to select the best-fitted model by a one-way analysis of variance (ANOVA). The selected model was further explained by a polynomial equation and different plots were produced using the software.

### 2.3. Production of AGN-PLHNPs

**Figure 1:**
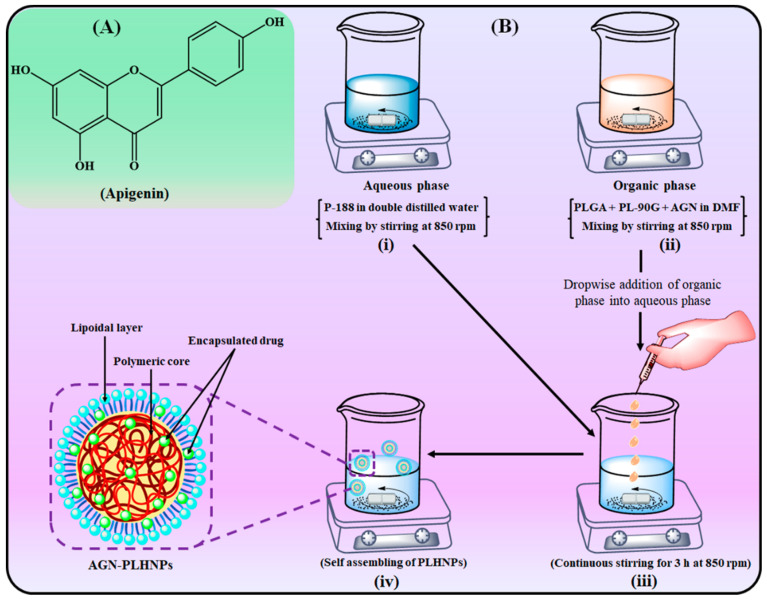
(**A**) Chemical structure of Apigenin ((**B**) **i**–**iv**) Schematic representation of preparation of AGN-PLHNPs single step nanoprecipitation method. (**i**). Aqueous surfactant solution (blue colour); (**ii**). Organic solution of AGN, PLGA, and PL-90G (pink colour); (**iii**). Addition of aqueous phase to organic phase with continuous stirring (sky colour); (**iv**). Formation of AGN-PLHNPs (green colour).

The AGN-PLHNPs were prepared with polymer (PLGA), phospholipid (PL-90G), and surfactant (P-188) by a single-step nanoprecipitation method [18,19], and a scheme for the preparation is illustrated in Figure 1B. The concentration of PLGA (F1), PL-90G (F2), and P-188 (F3) were taken as per the concentration suggested by the 33-BBD as summarized in Table 2. Briefly, accurately weighed quantity of AGN (20 mg), PLGA (7–11 mg/mL), and PL-90G (5–9 mg/mL) were dissolved in N, N-Dimethylformamide (2 mL, DMF) by using a magnetic stirrer (Remi, Mumbai, India) at room temperature to prepare the organic phase. Separately, P-188 (0.75–1.25%) was dissolved in double distilled water (8 mL) to prepare the aqueous phase at room temperature. Then, the aqueous phase was transferred drop by drop with the help of a syringe at 1 mL/min rate to the organic phase under the continuous stirring speed of 750 rpm. The resulting mixture was further stirred at the same speed for 3 h for self-assembling of the nanoparticles. After that, AGN-PLHNPs were dialyzed for 12 h against HPLC grade water to remove the organic phase Finally, the AGN-PLHNPs were taken in airtight glass vials and stored for further studies.

### 2.4. Characterization of AGN-PLHNPs

#### 2.4.1. Particle Evaluation

The particle size (PS), polydispersity index (PDI), and Zeta potential (ZP) of AGN-PLHNPs were analyzed by a zeta sizer (Nano ZS, Malvern Instruments, Worcestershire, UK). The samples were placed in the zeta cuvette and analyzed at room temperature at a scattering angle of 90°.

#### 2.4.2. Transmission Electron Microscopic (TEM) Analysis

The morphology of the optimized AGN-PLHNPs was observed under a transmission electron microscope (TEM; Morgagni 268D, Eindhoven, The Netherlands). The microscope was operated at 200 kV for point-to-point resolution. A drop of AGN-PLHNPs was dropped onto a copper grid with 300 mesh and stained negatively with 1% phosphotungstic acid. After this, the grid was air dried and visualized under the microscope.

#### 2.4.3. Entrapment Efficiency (%EE) and Loading Capacity (%LC)

The %EE and %LC of AGN-PLHNPs were quantified by an indirect method using centrifugation [20]. Briefly, AGN-PLHNPs were centrifuged for 20 min at 15,000 rpm with the help of a cooling centrifuge (Sigma 3K30, Sigma Laboratory, Germany). After this, the supernatant was taken and filtered with a membrane filter (0.2 µm pore size). Then, the unentrapped AGN was quantified with the help of a UV-Spectrophotometer (Shimadzu, UV-1601 model, Kyoto, Japan) at 268 nm $\lambda_{\max}$. The %EE and %LC were calculated by the following equations.

(1) $$\%\mathrm{EE}=\frac{\mathrm{Total}\;\mathrm{weight}\;\mathrm{of}\;\mathrm{AGN}\;-\mathrm{Free}\;\mathrm{AGN}\;}{\mathrm{Total}\;\mathrm{weight}\;\mathrm{of}\;\mathrm{AGN}}\times 100$$

(2) $$\%\mathrm{LC}=\frac{\mathrm{Total}\;\mathrm{weight}\;\mathrm{of}\;\mathrm{AGN}-\mathrm{Free}\;\mathrm{AGN}}{\mathrm{Total}\;\mathrm{weight}\;\mathrm{of}\;\mathrm{PLHNPs}}\times 100$$

### 2.5. Colloidal Stability

To evaluate the colloidal stability, the optimized AGN-PLHNPs (5 mL) were taken into glass vials and stored at 4 ± 1 °C, 25 ± 2 °C, and 40 ± 2 °C for 6 months in a stability chamber (KBF-240 model, Binder Gmbh, Tuttlingen, Germany) as per the ICH guide-line [20,21]. The change in the PS, PDI, ZP, and %EE of the optimized AGN-PLHNPs was noted after every 30th day for 6 months.

### 2.6. In Vitro AGN Release

The in vitro release of AGN from the optimized AGN-PLHNPs and AGN suspension was estimated using the dialysis bag method [22]. Before the experiment, the dialysis bag was activated as per the procedure suggested by Sigma Aldrich. After this, 2.5 mL of the formulation (~5 mg AGN) was taken in the dialysis bag and both ends were ligated with thread. Then, the formulation containing the dialysis bag was immersed in dissolution medium (500 mL). The experiment was performed at 37 ± 1 °C and 100 rpm stirring speed. At predefined times, 3 mL aliquots were taken and an equal volume (3 mL) of fresh medium was added to the dissolution medium to maintain the sink condition. The removed sample was filtered, diluted appropriately and the released quantity of AGN was determined by analyzing the absorbance of AGN with the help of a UV-Spectrophotometer at 268 nm [23]. The release of pure AGN was also determined by a similar procedure and the results were compared to evaluate the dissolution enhancement ability of the optimized AGN-PLHNPs. To analyze the kinetics of AGN release from the optimized AGN-PLHNPs, the release data (i.e., %CDR) were fitted in various mathematical models. The model which revealed the highest correlation coefficient (R^2^) was considered to be the best-fit model to analyze the release mechanism of AGN from the optimized AGN-PLHNPs [24].

### 2.7. Antioxidant Activity

The investigation, we assessed the antioxidant activity of the optimized AGN-PLHNPs by two methods, viz., a DPPH (2,2-diphenyl-1-picrylhydrazyl) assay and ABTS {2,2′-azino-bis (3-ethylbenzthiazoline-6-sulfonic acid)} assay and compared the results with free AGN solution.

#### 2.7.1. DPPH Assay

The DPPH assay was used to analyze the antioxidant potential of the optimized AGN-PLHNPs and free AGN was evaluated as per the reported protocol, with minor modifications [25]. For conducting this experiment, free AGN and the optimized AGN-PLHNPs were dis-solved in ethanol to make a 10 mg/mL concentration as stock solutions. A serial dilution from 20–100 µg/mL was prepared by diluting the stock solutions. The obtained solution was filtered through a 0.4 µm membrane filter to remove any residue present in the solution. After that, a 0.02% DPPH solution was prepared in ethanol and 0.5 mL of each concentration of free AGN, and the optimized solution was added to 125 μL of the DPPH solution. The resulting mixture was vortexed gently and was kept in a dark place for 1 h to complete the reaction. The change in the color from violet to colorless indicates the completion of the reaction. After 1 h, the absorbance was noted at 517 nm by a UV-Spectrophotometer. A low absorbance value indicates a high radical scavenging potential of the sample. In addition, a similar procedure was followed using DPPH solution as the control, and the results were compared. Finally, the % radical scavenging activity (%RSA) was calculated with the help of Equation (3).

(3) $${\%\mathrm{RSA}}_{\mathrm{DPPH}}=\frac{{\mathrm{Absorbance}}_{\mathrm{Control}}-{\mathrm{Absorbance}}_{\mathrm{Sample}}}{{\mathrm{Absorbance}}_{\mathrm{Control}}}\times 100$$

#### 2.7.2. ABTS Assay

The ABTS assay was used to analyze the antioxidant potential of the optimized AGN-PLHNPs and free AGN was evaluated as per the reported protocol with minor modifications [26]. For conducting this experiment, free AGN and the optimized AGN-PLHNPs were dis-solved in ethanol to make a 10 mg/mL concentration as stock solutions. A serial dilution from 20–100 µg/mL was prepared by diluting the stock solutions. The obtained solution was filtered through a 0.4 µm membrane filter to remove any residue present in the solution. After that, 0.1 mL of the sample at each concentration was added to 0.9 mL of the ABTS solution. The resulting mixture was stored in a dark place on a water bath at room temperature for 30 min to complete the reaction. After 30 min, the absorbance was noted at 734 nm by a UV-Spectrophotometer. In addition, a similar procedure was followed using the ABTS solution as a control, and the results were compared. Finally, the %RSA was calculated with the help of Equation (4).

(4) $${\%\mathrm{RSA}}_{\mathrm{ABTS}}=\frac{{\mathrm{Absorbance}}_{\mathrm{Control}}-{\mathrm{Absorbance}}_{\mathrm{Sample}}}{{\mathrm{Absorbance}}_{\mathrm{Control}}}\times 100$$

### 2.8. Cell Culture Studies

For cell culture studies, MCF-7, MDA-MB-231, MCF-10A, and 3T3 cell lines were used. All the cancer cells were procured from the National Centre for Cell Science (NCCS), Maharashtra, India. The cells were cultured in Dulbecco’s modified Eagle medium (DMEM) containing 100 mg/mL streptomycin + 10% fetal bovine serum + 100 U·mL^−1^ penicillin under specific environmental conditions. The cell culture experiments were performed when 80 to 90% growth of the cells was achieved [27].

#### 2.8.1. Cellular Uptake in Breast Cancer Cells

This experiment was performed to analyze the uptake potential of the developed PLHNPs by breast cancer cells by using Rhodamine B (Rh-B) dye as a fluorescent dye. Before the experiment, RhB-loaded PLHNPs were prepared by a procedure similar to that in Section 2.3. After this, both cells, i.e., MCF-7 and MDA-MB-21 cells with 1 × 10^5^ cell density, were seeded for 24 h in a 6-well plate over the glass coverslips. After 24 h, the culture medium was replaced with 2 mL fresh culture media containing free Rh-B, and RhB-loaded PLHNPs with 5 μgmL^−1^ Rh-B concentration and further incubated for 4 h. After 4 h of incubation, the cells were washed three times with PBS and the cover slip was fixed on the glass slide with 4% paraformaldehyde (4%). Finally, the cellular uptake of the cells was visualized with a fluorescence microscope (Olympus Inc., Tokyo, Japan) [28].

#### 2.8.2. Cytotoxicity Assay in Breast Cancer Cells

The cytotoxicity assay of both free AGN and the optimized AGN-PLHNPs was examined in MDA-MB-231 and MCF-7 cells by Methyl-thiazolyl-tetrazolium (MTT) assay [29]. Briefly, both MDA-MB-231 and MCF-7 cells were seeded in 96 well plates with a density of 1 × 10^5^ cells per well and incubated for 24 h. After confluence, both cancer cells were treated with different concentrations of free AGN, optimized AGN-PLHNPs, and blank PLHNPs and incubated in a CO2 incubator for 24 h, 48 h, and 72 h. Just after the completion of the incubation period, 25 μL of MTT dye (pre-pared in phosphate buffer saline; 0.5 mg/L concentration) was added to each well and plate and then all the plates are again incubated for 4 h to develop formazan crystals. After incubation of 4 h, excess culture media from each well was removed and formazan crystals were solubilized by adding 100 μL DMSO. Then plates are gently shaken on a shaker for 30 min for complete solubilization of crystals. After that, the optical density of soluble formazan was measured by a plate reader (Bio-Rad 680, Hercules, CA, USA) at 570 nm wavelength. In the end, the IC50 i.e., half of the maximum inhibitory concentrations of free AGN and the optimized AGN-PLHNPs was calculated at each time point with the help of GraphPad Prism (V.7) software (GraphPad, San Diego, CA, USA).

#### 2.8.3. Cytotoxicity Assay in Non-Neoplastic Cells

This study was performed using MCF-10A and 3T3 non-neoplastic cell lines following the same procedure discussed in Section 2.8.2 [30].

### 2.9. Statistical Analysis

All the experiments are conducted in triplicate and the results shown as mean ± standard deviation (SD). Results were analyzed statistically significance (*p* < 0.5) by one-way ANOVA followed by Student’s t-test with the help of GraphPad Prism version 7 (GraphPad, San Diego, CA, USA).

## 3. Results and Discussion

### 3.1. Optimization of AGN-PLHNPs by Box–Behnken Statistical Design

The results were obtained after the development of all the 15 formulations, and the value of each response, i.e., R_1_ (PS), R_2_ (%EE), and R_3_ (%CDR), was fitted in three different models, viz., linear, two-factor interaction (2FI) and quadratic models, in the software and analyzed by linear regression to select the best-fitted model. The statistical model that yielded the highest (nearest to 1) adjusted as well as predicted R^2^ was selected as the best-fitted model (Table 3). Further, significant terms of the best-fitted model were identified by ANOVA analysis on each response and the obtained results are presented in Table 4. When a model’s terms have a “*p*” value < 0.05, then it is considered statistically significant. As per the results, summarized in Table 3, the quadratic model has the highest adjusted and predicted R^2^ for all three responses and was selected as the best-fitted statistical model. Furthermore, the final quadratic polynomial equation for each response related to all the dependent factors in terms of coded factors was obtained after the model reduction. The quadratic polynomial equation obtained from the Design Expert^®^ software for all the responses is as follows:

R_1_ (PS) = +126.68 + 19.99F_1_ + 17.27F_2_ − 11.72F_3_ + 0.15F_1_F_2_ + 1.29F_1_F_3_ + 0.175F_2_F_3_ + 6.35F_1_² + 5.42F_2_² + 6.30F_3_² (5)

R_2_ (%EE) = +77.84 + 5.76F_1_ + 6.06F_2_ + 1.69F_3_ − 0.61F_1_F_2_ − 1.85F_1_F_3_ + 1.74F_2_F_3_ − 3.32F_1_² − 4.17F_2_² − 5.36F_3_² (6)

R_3_ (%CDR) = +82.57 − 6.40F_1_ − 3.69F_2_ + 4.58F_3_ − 0.81F_1_F_2_ − 0.285F_1_F_3_ − 0.2375F_2_F_3_ − 0.0387F_1_² − 0.7162F_2_² − 0.4963F_3_² (7)

The 3D surface, contour, perturbation, and predicted vs. actual plots generated from the design were utilized to analyze the effect of independent factors (i.e., F_1_, F_2_, and F_3_) on responses (i.e., R_1_, R_2_, and R_3_). In addition, the polynomial equations of each response were further utilized to examine the effect of all three factors on each response.

#### 3.1.1. Effect on R_1_ (PS)

**Figure 2:**
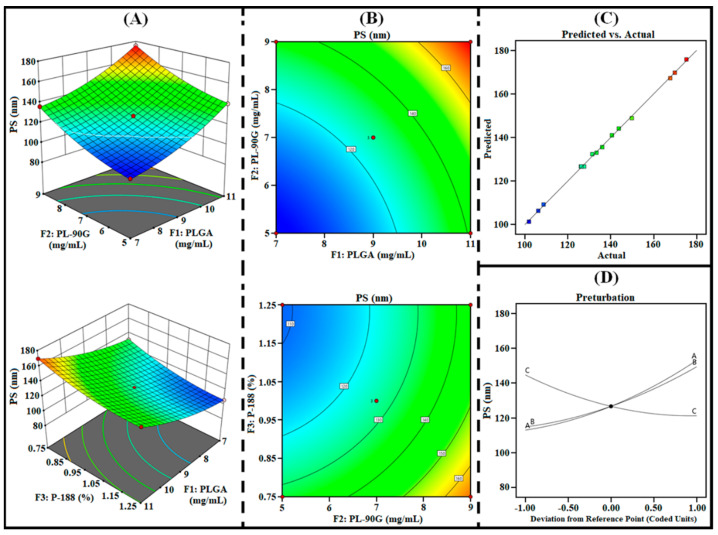
Three-dimensional surface plots (**A**), contour plots (**B**), predicted vs. actual plot (**C**), and perturbation plot (**D**), highlighting the effects of independent factors (F_1_ = PLGA; F2 = PL-90G; and F3 = P-188) on R_1_ (PS) of AGN-PLHNPs.

The size of the PLHNPs should be small enough for enhanced cellular internalization and cytosolic release of the entrapped drug into the cancer cells. As represented in Table 2, the PS of AGN-PLHNPs was found between 101.93 nm to 175.26 nm, indicating the formation of PLHNPs with small PS. As per the ANOVA analysis, all the three factors i.e., F1, F2, as well as F3 significantly (*p* < 0.0001) influence the PS (R1). In addition, the polynomial Equation (5). and different statistical plots as illustrated in Figure 2 represent a significant effect of all independent factors on PS (R1). The PS of the AGN-PLHNPs was directly proportional to the concentration of PLGA (F1). A significant increase in the PS of AGN-PLHNPs attributed to increasing in the viscosity of organic phase on increasing PLGA concentration from 7 mg/mL to 11 mg/mL. The high viscosity of the organic phase significantly decreases the shear efficiency that is responsible for the reduction of PS during the development of PLHNPs [31]. In addition, the high viscosity of the organic phase significantly reduces the diffusion rate from organic to aqueous phase by Ostwald ripening phenomenon that further increases the PS of the AGN-PLHNPs [32]. Similarly, a gradual increase in PL-90G (F2) from 5 mg/mL to 9 mg/mL gradually increases the PS of the AGN-PLHNPs. A gradual enhancement in PS of AGN-PLHNPs attributed to increase in the viscosity of the organic solution that directly increases the interfacial tension of aqueous/organic interface. High interfacial tension can favor lipid coalescence that leads to the production of AGN-PLHNPs with greater PS [33]. However, the third factor P-188 (F3) represents a negative effect on the PS of the AGN-PLHNPs on increasing the P-188 concentration from 0.75% to 1.25% *w*/*v*. The PS of the AGN-PLHNPs significantly decreases on increasing P-188 concentration attributed to the reduction in the interfacial tension at the interface that greatly enhances the emulsification of polymer and lipid within the system. High emulsification of polymer and lipids takes place during the development of AGN-PLHNPs resulting small PS [34].

#### 3.1.2. Effect on R_2_ (%EE)

**Figure 3:**
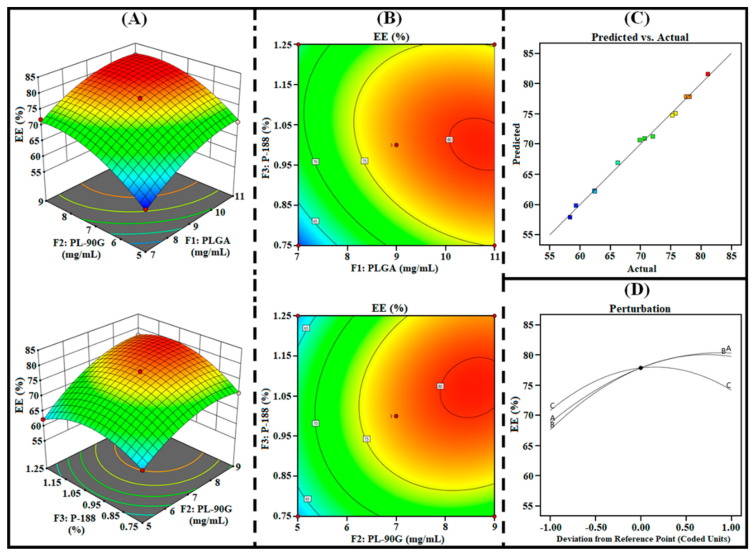
Three-dimensional surface plots (**A**), contour plots (**B**), predicted vs. actual plot (**C**), and perturbation plot (**D**), highlighting the effects of independent factors (F_1_ = PLGA; F2 = PL-90G; and F3 = P-188) on R_2_ (%EE) of AGN-PLHNPs.

High %EE is one of the major advantages of developing PLHNPs. The %EE of the PLHNPs should be high to achieve desired therapeutic efficacy. As represented in Table 2, the %EE of AGN-PLHNPs was found between 58.35 % to 81.14 % suggesting the development of PLHNPs with optimal loading capacity. As per the ANOVA analysis, all the three factors i.e., F1, F2, as well as F3 significantly (*p* < 0.0001) influence the %EE (R2). In addition, the polynomial Equation 6. and different statistical plots as illustrated in Figure 3 represent a significant effect of all independent factors on %EE (R2). A gradual increment in the PLGA (F1) concentration from 7 mg/mL to 11 mg/mL during the development of AGN-PLHNPs significantly increases the %EE. This increment in the %EE was attributed to an increase in the space for AGN encapsulation in the hybrid matrix by increasing PLGA concentration that provides AGN-PLHNPs with significantly greater %EE [35]. A similar effect on %EE was also observed on increasing the concentration of PL-90G (F2) from 5mg/mL to 9 mg/mL. Gradual increment in the %EE attributed to the significant increase in the viscosity of the organic phase on increasing PL-90G concentration that resulted in rapid solidification during the development of AGN-PLHNPs. Rapid solidification of lipids significantly prevents the drug diffusion from the hybrid matrix and produces PLHNPs with higher %EE [36]. Furthermore, on the initial increment in P-188 (F3) concentration from 0.75% *w*/*v* to 1.0% *w*/*v*, the %EE of the AGN-PLHNPs increases gradually. This increment in the %EE was attributed to the higher emulsification of PLGA and PL-90G within the system during the formulation development that provides the AGN-PLHNPs with a high %EE. However, with the increment in the concentration of P-188 from 1.0% *w*/*v* to 1.25% *w*/*v*, the %EE of the AGN-PLHNPs decreases significantly. This reduction in the %EE was attributed to the increment in the portioning of the encapsulated AGN from the internal to external phase that increases the leakage of encapsulated drug from the hybrid matrix and produces AGN-PLHNPs with a low %EE [37].

#### 3.1.3. Effect on R_3_ (%CDR)

**Figure 4:**
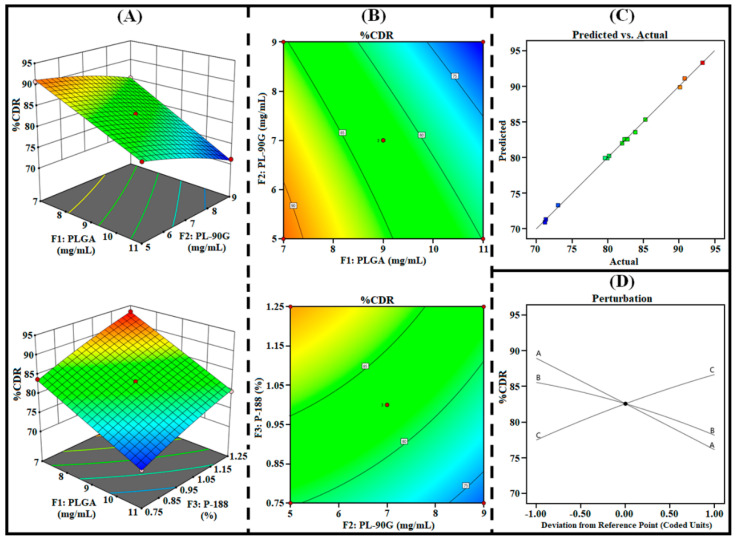
Three-dimensional surface plots (**A**), contour plots (**B**), predicted vs. actual plot (**C**), and perturbation plot (**D**), highlighting the effects of independent factors (F_1_ = PLGA; F2 = PL-90G; and F3 = P-188) on R_3_ (%CDR) of AGN-PLHNPs.

The release of entrapped drugs from the hybrid matrix of PLHNPs is an important parameter in achieving desired therapeutic efficacy and it is directly related to the PS that significantly modifies overall therapeutic potential [38]. The nanoparticles with small PS represent the fast release of encapsulated drugs due to the high surface area [39]. Conversely, the nanoparticles with large PS represent a comparatively slow re-lease of the encapsulated drug due to lesser surface area for diffusion [40]. As represented in Table 2, the %CDR of AGN-PLHNPs was found between 71.21% to 93.31% suggesting the development of PLHNPs with controlled release characteristics. As per the ANOVA analysis, all the three factors i.e., F1, F2, as well as F3 significantly (*p* < 0.0001) influence the %CDR (R3). In addition, the polynomial Equation 7. and different statistical plots are illustrated in Figure 4. It represents a significant effect of all independent factors on %CDR (R3). Gradual increase in the PLGA (F1) and PL-90G (F2) significantly decreases the %CDR of AGN-PLHNPs. A significant reduction in the %CDR on increasing PLGA concentration was attributed to the development of a thick and compact hybrid matrix of PLHNPs that results in slow diffusion of the drug. In addition, increment in the PLGA concentration produces AGN-PLHNPs with large PS which provides relatively less surface area that is also a major factor for slow drug re-lease [31]. Similarly, a gradual increment in the lipid (PL-90G; F2) concentration de-creases the %CDR of AGN. The reduction in %CDR with increment in the PL-90G at-tributed to an enhancement in the viscosity that produces PLHNPs with large PS. As stated earlier, the nanoparticles with large PS show relatively less %CDR [33]. Conversely, the %CDR of PLHNPs was increased on increasing P-188 (F3) concentration. This increment in the %CDR attributed to the production of AGN-PLHNPs with small PS on increasing P-188 concentration [18].

#### 3.1.4. Optimized AGN-PLHNPs Selection

Among the 15 compositions obtained from the 3^3^-BBD that fulfill the criteria by “trading off” all responses with a small PS, a high %EE and high %CDR were chosen as the optimized composition. The AGN-PLHNPs prepared with 9 mg/mL of PLGA (polymer), 7 mg/mL of PL-90G (lipid), and 1% *w*/*v* of P-188 (surfactant) fulfilled the criteria for the development of optimized AGN-PLHNPs. Therefore, three AGN-PLHNPs were prepared with the optimized composition for further studies. The optimized AGN-PLHNPs revealed an average PS, %EE, and %CDR of 125.73 ± 5.57 nm, 77.43 ± 3.62%, and 82.37 ± 4.12, respectively.

### 3.2. AGN-PLHNPs Characterization

#### 3.2.1. PS, PDI, and ZP Determination

**Figure 5:**
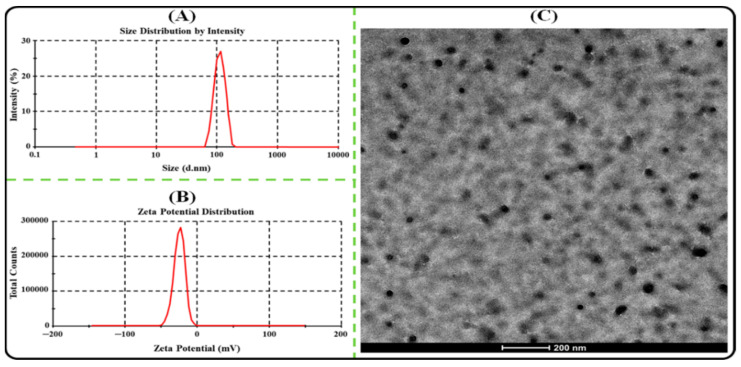
(**A**) Particle size and distribution; (**B**) Zeta potential distribution; (**C**) TEM micrograph of the optimized AGN-PLHNPs.

The PS of the PLHNPs should be <200 nm for effective tumor-targeted drug de-livery. Nanoparticles with PS < 200 nm promotes cellular internalization and drug ac-cumulation at the tumor site [41]. The optimized AGN-PLHNPs exhibited an average PS of 125.73 ± 5.57 nm as represented in Figure 5A. The PDI represents the uniformity of PS in the nanoparticles system. The PDI value of the nanoparticles < 0.3 revealed good homogeneity [42], and exhibited an average PDI of 0.18 ± 0.02. The ZP is a measure of the charge on the surface of nanoparticles and plays a significant role in the colloidal stability of the nanoparticles [43]. A higher positive, as well as negative ZP, dis-plays strong repulsion among the particles indicating high colloidal stability [44]. The optimized AGN-PLHNPs exhibited an average ZP of –26.71 ± 1.93 mV as depicted in Figure 5B. A higher negative ZP on AGN-PLHNPs was attributed to presence of carboxyl groups of PLGA as well as P-188 in the formulation [45].

#### 3.2.2. TEM Analysis

The TEM micrograph of AGN-PLHNPs is depicted in Figure 5C. As per Figure 5C, the optimized AGN-PLHNPs were spherical and uniformly distributed as well as separated from each other. Furthermore, the size of AGN-PLHNPs was found to be in the range of <150 nm. Therefore, the result of TEM analysis also corroborated the result obtained from the zeta sizer.

#### 3.2.3. %EE and %LC

For the successful development of PLHNPs, the %EE and %LC should be optimal to achieve the desired therapeutic efficacy. The optimized AGN-PLHNPs revealed an %EE and %LC of 77.43 ± 3.62%% and 7.56 ± 0.27%, respectively. Overall, optimum and acceptable %EE and %LC values were obtained for AGN-PLHNPs because of the hybrid matrix.

### 3.3. Colloidal Stability

**Figure 6:**
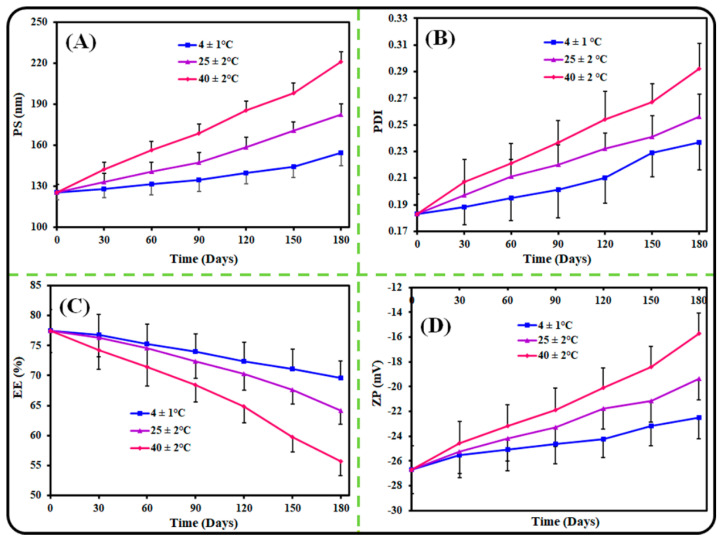
Colloidal stability of the optimized AGN-PLHNPs under different environmental conditions and different time intervals: (**A**) PS, (**B**) PDI, (**C**) %EE, and (**D**) %CDR.

This study was conducted to understand the colloidal stability of the optimized AGN-PLHNPs in different environmental conditions since the environmental conditions significantly affect the stability of pharmaceutical preparations. To analyze the colloidal stability, the optimized AGN-PLHNPs were stored under three different storage conditions, i.e., 4 ± 1 °C, 25 ± 2 °C, and 40 ± 2 °C, for 6 months, and the observed results are shown in Figure 6. As per the results, the optimized AGN-PLHNPs represented better stability at 4 ± 1 °C and 25 ± 2 °C and revealed only insignificant (*p* > 0.05) changes in the PS, PDI, ZP, and %EE even after 6 months. However, the optimized AGN-PLHNPs revealed significant (*p* < 0.05) changes in the PS, PDI, ZP, and %EE at 40 ± 2 °C. At elevated temperatures, i.e., 40 ± 2 °C, the PS and PDI increase significantly due to the swelling behavior of the polymer [46]. In addition, the ZP of the optimized AGN-PLHNPs was decreased upon storage at an elevated temperature due to the breakdown of the polymeric network of the PLHNPs. Furthermore, %EE decreased due to the leakage of the drug from the hybrid matrix of the nanoparticles upon the storage at an elevated temperature [20].

### 3.4. In Vitro AGN Release

**Figure 7:**
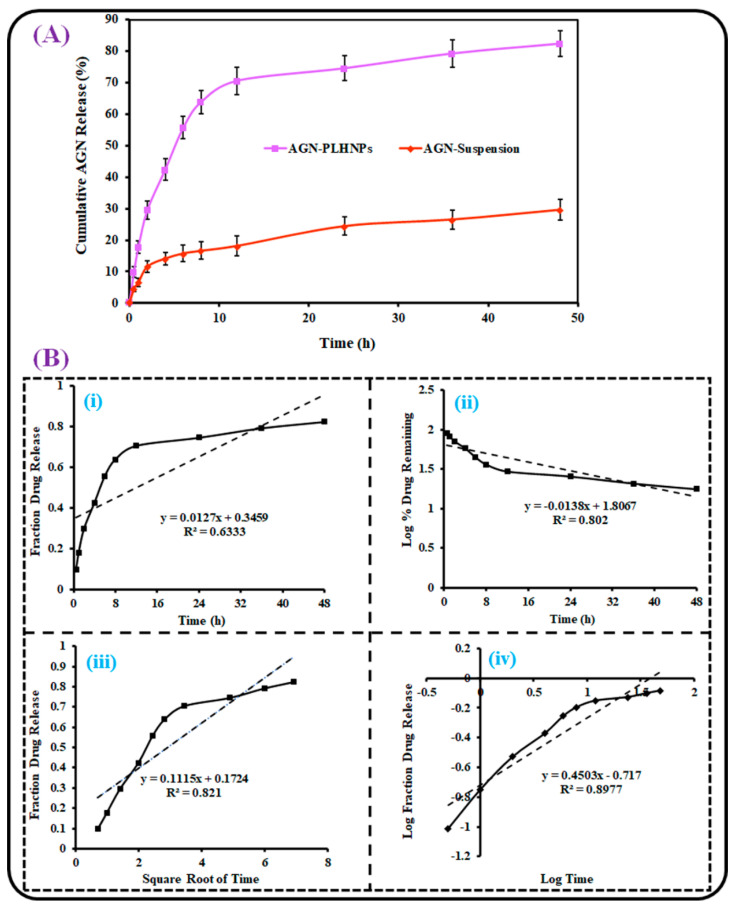
(**A**) AGN release profiles from the optimized AGN-PLHNPs and AGN suspension and (**B**) various kinetic models: (**i**) Zero-order model, (**ii**) First-order model, (**iii**) Higuchi matrix model, and (**iv**) Korsmeyer–Peppas model for optimized AGN-PLHNPs to understand the mechanism.

The release behavior of AGN from AGN-PLHNPs and the AGN suspension was assessed for 48 h using a dialysis membrane. The comparative drug release profiles of AGN-PLHNPs and the AGN suspension are depicted in Figure 7A. As expected, the AGN suspension revealed only 29.65 ± 3.21% AGN release. The poor release of AGN from the conventional suspension is ascribed to the poor solubility of free AGN in the dissolution media. On the other hand, the optimized AGN-PLHNPs revealed a biphasic AGN release profile. For the initial 4 h, the optimized AGN-PLHNPs showed the fast release (42.31 ± 3.26%) of AGN from the PLHNPs and, after this, a sustained release for up to 48 h (82.37 ± 4.12%). The fast release in the initial 4 h was attributed to the diffusion of AGN adsorbed on the PLHNP’s surface. In addition, the fast release was also achieved because of the small PS of AGN-PLHNPs [43]. After 4 h, a sustained release was achieved from the optimized AGN-PLHNPs because of the slow diffusion of the drug encapsulated in the inner hybrid matrix of PLHNPs. Furthermore, the crystalline and hydrophobic characteristics of PLGA are also responsible for the sustained release of AGN from the PLHNPs [47].

To analyze the kinetics of AGN release from the optimized PLHNPs, the release data of the optimized AGN-PLHNPs was fitted in various release kinetic models and the obtained outputs are represented in Figure 7B. Korsmeyer-Peppas model revealed the maximum R2 (closest to 1) with R2 = 0.8977, and selected to be the best-fitted model and the release exponent (“n”) from this model was calculated. The “n” value from the model was observed to be 0.195 and was less than 0.5. Therefore, it was suggested that the mechanism of AGN release from the PLHNPs was the “Fickian diffusion” [48].

### 3.5. Antioxidant Activity

**Figure 8:**
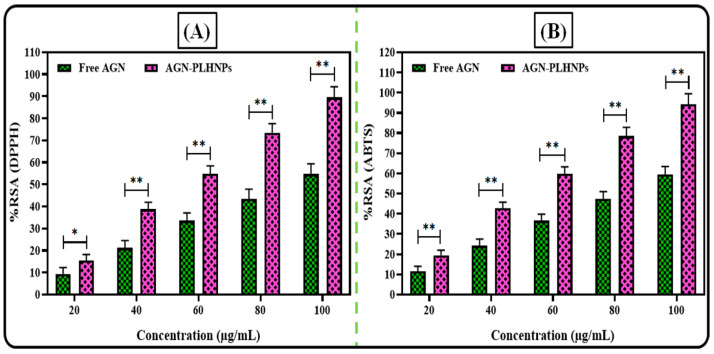
Comparative radical scavenging activity (RSA) of the optimized AGN-PLHNPs and free AGN through (**A**) DPPH assay and (**B**) ABTS assay. * Significant to free AGN and ** highly significant to free AGN. (*p* < 0.05 considered as significant difference).

As stated earlier, AGN is a lipophilic phytochemical known for its antioxidant activity, but its therapeutic efficacy is restricted due to its high lipophilicity. The pre-pared AGN encapsulated PLHNPs showed higher solubility and dissolution. The antioxidant profiles from both DPPH and ABTS assays are depicted in Figure 8.

#### 3.5.1. DPPH Assay

The comparative antioxidant profiles of free AGN and the optimized AGN-PLHNPs by DPPH assay are depicted in Figure 8A. As per the results, both free AGN as well as AGN-PLHNPs represented concentration-dependent %RSA. As expected, the optimized AGN-PLHNPs represented higher %RSA compared to the free AGN. At the concentration of 100 µg/mL, the optimized AGN-PLHNPs and free AGN exhibited the %RSA of 89.51 ± 4.83% and 54.72 ± 3.67% respectively. Overall, the optimized AGN-PLHNPs exhibited significantly enhanced (*p* < 0.05) %RSA when compared with free AGN. A significant improvement in the antioxidant activity of AGN after encapsulation into the PLHNPs was found due to the enhancement in solubility as well as controlled diffusion of the encapsulated drug from the hybrid nanoparticles [49].

#### 3.5.2. ABTS Assay

The comparative antioxidant profiles of AGN and the optimized AGN-PLHNPs by ABTS assay are shown in Figure 8B. As per the results, both free AGN as well as AGN-PLHNPs represented concentration-dependent %RSA as similar to the DPPH assay. At the concentration of 100 µg/mL, the optimized AGN-PLHNPs and free AGN exhibited the %RSA of 94.27 ± 5.16% and 59.34 ± 4.12%, respectively. Overall, the optimized AGN-PLHNPs exhibited significantly enhanced (*p* < 0.05) %RSA when compared with free AGN. The enhancement in findings from optimized AGN-PLHNPs due to the improvement in the solubility as well as the controlled diffusion of the encapsulated drug from the hybrid nanoparticles [50].

### 3.6. Cell Culture Studies

#### 3.6.1. Cellular Uptake Study

**Figure 9:**
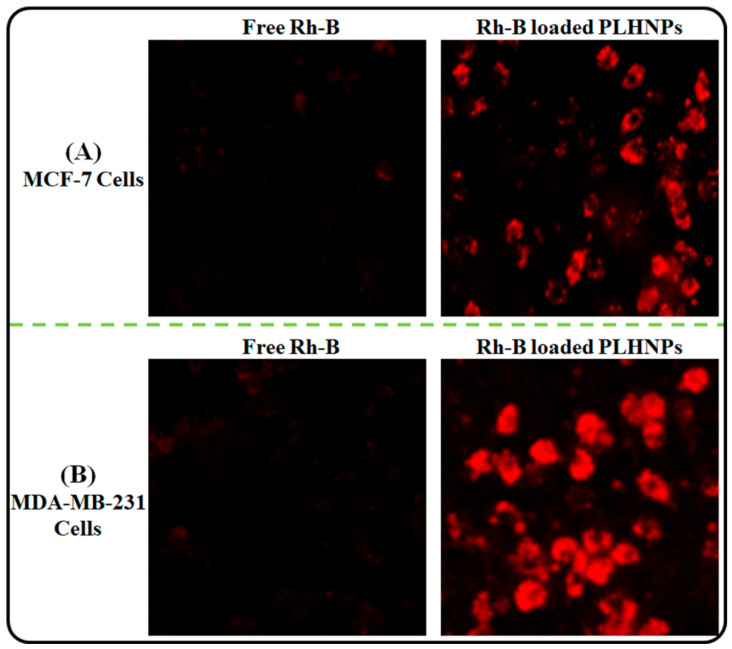
Fluorescence microscopic image illustrating the cellular uptake of free Rh-B and Rh-B loaded PLHNPs in (**A**) MCF-7 cells and (**B**) MDA-MB-231 cells.

The uptake of the nanoparticles by the cancer cells should be high enough to achieve better therapeutic efficacy. The cellular uptake of PLHNPs in MCF-7 and MDA-MB-231 cells are depicted in Figure 9. As per results, the PLHNPs exhibited higher cellular internalization in both MCF-7, as well as MDA-MB-231 cells compared to the free Rh-B solution. A better uptake of RhB-loaded-PLHNPs in both cells ascribed to the small PS (<200 nm). The small PS of PLHNPs offers a large surface area for cellular uptake in cancer cells [51]. Furthermore, the lipid present on the surface and matrix of PLHNPs might have facilitated the internalization of the PLHNPs through interaction among the lipids of the cell membranes [52].

#### 3.6.2. Cytotoxicity Assay in Breast Cancer Cells

**Figure 10:**
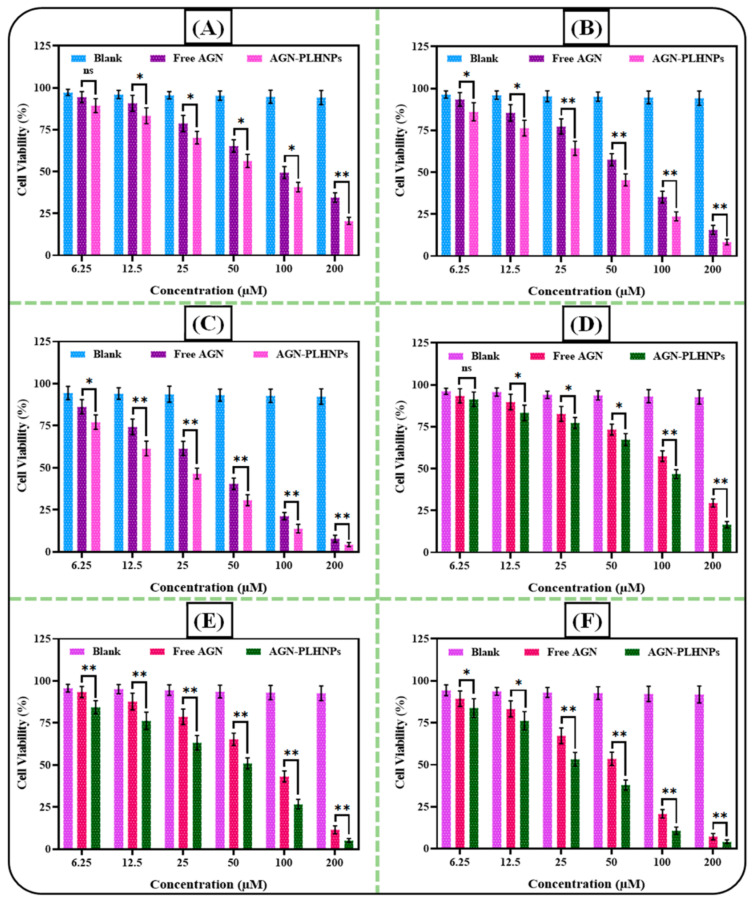
Cytotoxicity profiles of free AGN and AGN-PLHNPs against: (**A**) MCF-7 cells after 24 h; (**B**) MCF-7 cells after 48 h; (**C**) MCF-7 cells after 72 h; (**D**) MDA-MB-231 cells after 24 h; (**E**) MDA-MB-231 cells after 48 h; and (**F**) MDA-MB-231 cells after 72 h. * and ** indicates significant and highly significant difference between Free AGN and AGN-PLHNPs, (*p* < 0.05 considered significant), ns—non significant.

The cytotoxic profiles of the optimized AGN-PLHNPs and free AGN against MCF-7 and MDA-MB-231 breast cancer cell lines at 24 h, 48 h, and 72 h are depicted in Figure 10A–F. As per the results, blank PLHNPs revealed no toxicity against both the breast cancer cells and it can be inferred that the PLHNPs alone are non-toxic and safe for drug delivery. On the other hand, the optimized AGN-PLHNPs and free AGN revealed concentration as well as the time-dependent cytotoxicity against both breast cancer cells. At almost every concentration and time point (i.e., 24 h, 48 h as well as 72 h), the optimized AGN-PLHNPs represented significantly greater (*p* < 0.05) cytotoxicity in comparison to the free AGN against both cancer cells.

**Figure 11:**
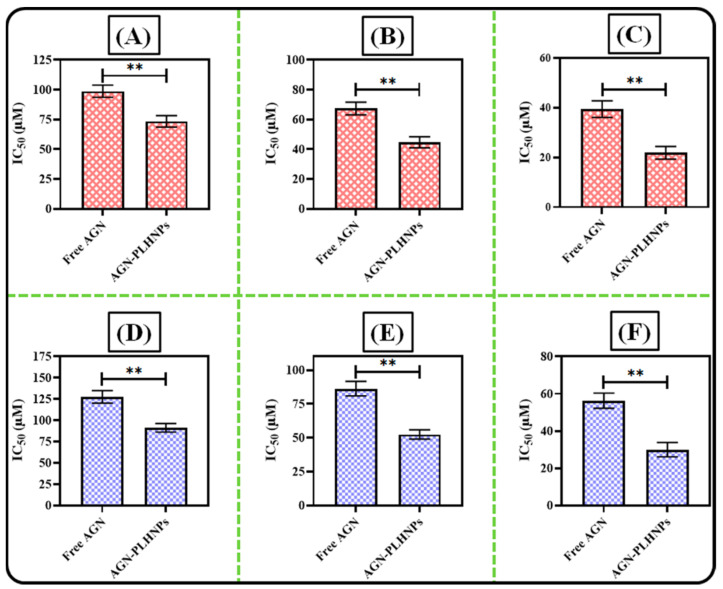
IC_50_ values of free AGN and AGN-PLHNPs against: (**A**) MCF-7 cells after 24 h; (**B**) MCF-7 cells after 48 h; (**C**) MCF-7 cells after 72 h; (**D**) MDA-MB-231 cells after 24 h; (**E**) MDA-MB-231 cells after 48 h; and (**F**) MDA-MB-231 cells after 72 h. ** indicates significant difference between Free AGN and AGN-PLHNPs, (*p* < 0.05 considered significant).

The IC_50_ value of free AGN and the optimized AGN-PLHNPs against both MCF-7 as well as MDA-MB-231 breast cancer cells is represented in Figure 11A–F. After 24 h of the incubation time, the IC50 of the optimized AGN-PLHNPs and free AGN was observed to be 73.47 ± 4.82 µM and 98.72 ± 5.12 µM respectively against MCF-7 cells (Figure 11A). After 48 h of the incubation period, the IC50 value of the optimized AGN-PLHNPs and free AGN was observed to be 44.63 ± 3.72 µM and 67.37 ± 4.26 µM respectively against MCF-7 cells (Figure 11B). While after 72 h of incubation time, the IC50 of the optimized AGN-PLHNPs and free AGN was observed to be 21.86 ± 2.53 µM and 39.45 ± 3.37 µM respectively against MCF-7 cells (Figure 11C). On the other hand, after an incubation period of 24 h, the optimized AGN-PLHNPs and free AGN were observed to be 91.17 ± 5.07 µM and 127.56 ± 7.38 µM respectively against MDA-MB-231 cells (Figure 11D). After 48 h of the incubation period, the IC50 value of the optimized AGN-PLHNPs and free AGN was observed to be 52.38 ± 3.47 µM and 86.24 ± 5.36 µM respectively against MDA-MB-231 cells (Figure 11E). While after 72 h of incubation, the IC50 of the optimized AGN-PLHNPs and free AGN was observed to be 30.47 ± 2.87 µM and 56.27 ± 4.07 µM respectively (Figure 11F). This enhanced cyto-toxicity of the optimized AGN-PLHNPs against both cancer cells was attributed to the small PS that provides higher surface area for exposure to the cancer cells. The slow and controlled release of encapsulated drugs from the hybrid matrix of the nanoparticles also helps to get better activity [53,54].

#### 3.6.3. Cytotoxicity Assay in Non-Neoplastic Cells

**Figure 12:**
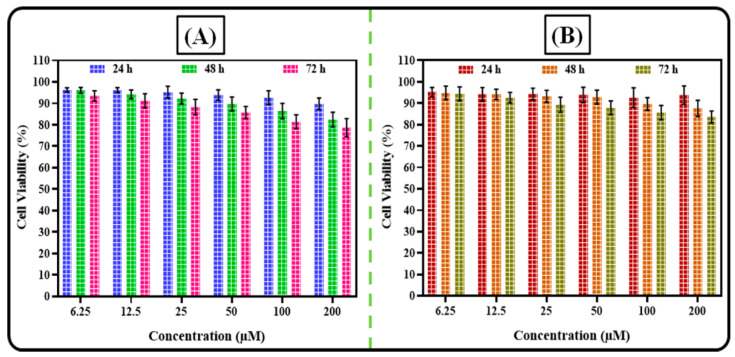
Cytotoxicity profiles of the optimized AGN-PLHNPs against (**A**) MCF-10A and (**B**) 3T3 non-neoplastic cells after 24 h, 48 h, and 72 h.

The most anticancer drugs show cytotoxicity against normal cells. The safety potential of optimized AGN-PLHNPs and the free AGN was analyzed against MCF-10A and 3T3 non-neoplastic cells. The results of the cytotoxic potential of free AGN and AGN-PLHNPs in both cells are depicted in Figure 12. Generally, >75% cell viability up on treatment with nanoparticles are considered as safe and non-cytotoxic to the non-neoplastic cells [55]. As per the results, the optimized AGN-PLHNPs exhibited less than 25% killing of both non-neoplastic cells after 72 h. As illustrated in Figure 12A, after treatment of the optimized AGN-PLHNPs, the cell viability of MCF-10A cells was observed to be 89.78 ± 2.73%, 82.45 ± 3.43%, and 78.69 ± 4.23%, respectively after 24 h, 48 h, and 72 h of study. While as per Figure 12B, after treatment of the optimized AGN-PLHNPs, the cell viability of 3T3 cells was observed to be 93.78 ± 4.33%, 87.61 ± 3.75%, and 83.61 ± 2.79% respectively after 24 h, 48 h, and 72 h of study. Therefore, this study revealed the safety of prepared AGN-PLHNPs.

## 4. Conclusions

In this investigation, AGN-PLHNPs prepared by a single-step nanoprecipitation technique and further optimized them by 33-BBD. The optimized AGN-PLHNPs revealed small PS (<130 nm), low PDI (<0.2), optimum %EE (>75%), and positive ZP (>±25). The optimized AGN-PLHNPs revealed good colloidal stability at low temperature as well as room temperature conditions. The encapsulation of AGN into PLHNPs improved the dissolution and represents sustained-release up to 48 h. Evaluation of antioxidant potential by both DPPH as well as ABTS revealed a higher %RSA com-pared to the free drug. Furthermore, the optimized AGN-PLHNPs exhibited dose as well as time-dependent cytotoxicity against MCF-7 and MDA-MB-231. Therefore, the in vitro results suggests that the optimized AGN-PLHNPs found to be an alternative delivery system with prolonged release as well as enhanced cell viability. The encouraging in-vitro results needs to be further evaluated for in-vivo study on suitable animal model.

## Figures and Tables

**Table 1:** Selected independent and dependent variables used to optimize AGN-PLHNPs by the 3^3^-BBD.

Independent Variables	Low (−1)	Medium (0)	High (+1)
F_1_ = Concentration of PLGA (mg/mL)	7	9	11
F_2_ = Concentration of PL-90G (mg/mL)	5	7	9
F_3_ = Concentration of P-188 (%)	0.75	1.00	1.25
Dependent variables	Goal		
R_1_ = Particle size (PS; nm)	Minimize		
R_2_ = Entrapment efficiency (EE; %)	Maximize		
R_3_ = Cumulative drug release (CDR; %)	Maximize		

**Table 2:** Composition of AGN-PLHNPs obtained from the 3^3^-BBD, with actual and predicted experimental values of their respective responses.

Independent Variables				Dependent Variables			Zeta Potential (mV)	PDI
Runs	F_1_ (PLGA; mg/mL)	F_2_ (PL-90G; (mg/mL)	F_3_ (P-188; % *w*/*v*)	R_1_ (PS; nm)	R_2_ (EE; %)	R_3_ (CDR; %)		
F1	7	9	1	136.03	72.04	85.29	−20.3	0.21
F2	11	5	1	140.56	69.89	79.96	−22.9	0.19
F3	11	9	1	175.26	81.14	71.21	−23.5	0.18
F4	7	7	1.25	106.24	66.24	93.31	−20.3	0.17
F5	11	7	0.75	169.85	75.78	71.33	−16.2	0.19
F6	9	5	1.25	108.74	62.45	90.14	−19.6	0.22
F7	7	5	1	101.93	58.35	90.8	−22.6	0.19
F8	7	7	0.75	131.39	59.37	83.88	−17.4	0.23
F9	9	9	1.25	143.76	77.72	82.03	−15.8	0.19
F10	11	7	1.25	149.84	75.27	79.62	−16.9	0.23
F11	9	7	1	126.34	76.87	81.78	−25.3	0.17
F12	9	7	1	125.62	78.45	82.76	−24.8	0.19
F13	9	5	0.75	133.39	62.39	80.21	−20.1	0.22
F14	9	9	0.75	167.71	70.68	73.05	−18.5	0.18
F15	9	7	1	126.07	77.11	82.23	−24.5	0.19

**Table 3:** Statistical summary of the applied model of 3^3^-BBD on R_1_ (PS), R_2_ (%EE), and R_3_ (%CDR).

Model	R^2^	Adjusted R^2^	Predicted R^2^	SD	Desirability	Remark
Response (R_1_; PS in nm)						
Linear	0.9486	0.9345	0.9293	5.74	–	–
2F1	0.9495	0.9137	0.8972	6.67	–	–
Quadratic	0.9993	0.9980	0.9911	1.01	0.95	Suggested
Response (R_2_; EE in %)						
Linear	0.7301	0.6565	0.5977	4.42	–	–
2F1	0.7645	0.5878	0.4842	4.85	–	–
Quadratic	0.9958	0.9881	0.9350	0.8223	0.83	Suggested
Response (R_3_; CDR in %)						
Linear	0.9895	0.9866	0.9798	0.7632	–	–
2F1	0.9947	0.9907	0.9815	0.6356	–	–
Quadratic	0.9990	0.9972	0.9860	0.3483	0.88	Suggested

**Table 4:** ANOVA data obtained after regression analysis and lack of fit tests for the best-fitted quadratic model for R_1_ (PS), R_2_ (%EE), and R_3_ (%CDR).

Model	Source	R_1_ (PS in nm)	R_2_ (EE in %)	R_3_ (CDR in %)
Regression analysis				
Quadratic	Sum of Squares	7038.21	794.48	609.69
	df	9	9	9
	Mean Square	782.02	88.28	67.74
	F-Value	769.73	130.55	558.46
	*p*-value, Prob > F	<0.0001	<0.0001	<0.0001
	Remark	Suggested, significant		
Lack of fit tests				
Quadratic	Sum of Squares	3.72	3.22	0.5223
	df	3	3	3
	Mean Square	1.24	1.07	0.1741
	F-Value	1.82	13.09	4.14
	*p*-value, Prob > F	0.3732	0.0718	0.2008
	Remark	Suggested, not significant		

## Data Availability

All the data is included in the current manuscript.

## Abbreviations

AGN = Apigenin; CDR = Cumulative drug release; EE = Entrapment efficiency; LC = Loading capacity; P-188 = Poloxamer 188; PBS = Phosphate buffer saline; PDI = Polydispersity index; PL-90G = Phospholipon 90G; PLGA = Poly D,L-lactic-co-glycolic acid; PLHNPs = Polymer–lipid hybrid nanoparticles; PS = Particle size; Rh-B = Rhodamine B; TEM = Transmission electron microscopy; ZP = Zeta Potential.
